# Network-Based Interpretation of Diverse High-Throughput Datasets through the Omics Integrator Software Package

**DOI:** 10.1371/journal.pcbi.1004879

**Published:** 2016-04-20

**Authors:** Nurcan Tuncbag, Sara J. C. Gosline, Amanda Kedaigle, Anthony R. Soltis, Anthony Gitter, Ernest Fraenkel

**Affiliations:** Department of Biological Engineering, Massachusetts Institute of Technology, Cambridge, Massachusetts, United States of America; UCSD, UNITED STATES

## Abstract

High-throughput, ‘omic’ methods provide sensitive measures of biological responses to perturbations. However, inherent biases in high-throughput assays make it difficult to interpret experiments in which more than one type of data is collected. In this work, we introduce Omics Integrator, a software package that takes a variety of ‘omic’ data as input and identifies putative underlying molecular pathways. The approach applies advanced network optimization algorithms to a network of thousands of molecular interactions to find high-confidence, interpretable subnetworks that best explain the data. These subnetworks connect changes observed in gene expression, protein abundance or other global assays to proteins that may not have been measured in the screens due to inherent bias or noise in measurement. This approach reveals unannotated molecular pathways that would not be detectable by searching pathway databases. Omics Integrator also provides an elegant framework to incorporate not only positive data, but also negative evidence. Incorporating negative evidence allows Omics Integrator to avoid unexpressed genes and avoid being biased toward highly-studied hub proteins, except when they are strongly implicated by the data. The software is comprised of two individual tools, Garnet and Forest, that can be run together or independently to allow a user to perform advanced integration of multiple types of high-throughput data as well as create condition-specific subnetworks of protein interactions that best connect the observed changes in various datasets. It is available at http://fraenkel.mit.edu/omicsintegrator and on GitHub at https://github.com/fraenkel-lab/OmicsIntegrator.

This is a *PLOS Computational Biology* Software paper.

## Introduction

High-throughput technologies are now able to provide comprehensive and quantitative measurements of molecular changes in response to perturbations or disease. Measurements of the transcriptome, epigenome, proteome, etc. serve to complete the opaque picture of the many active pathways and processes in cellular systems. However, no single dataset fully captures all aspects of cellular activity in a given experimental setting. For example, transcriptional datasets allow us to see which genes are up- or down-regulated relative to a control state, but do not provide information about post-translational modifications that are critical for signaling. In addition, high-throughput datasets often contain many ‘hits’ (i.e. species that change significantly between conditions in a given omic dataset) that either lie in unexpected pathways [1,2] or fail to map to any existing canonical pathways [1–5]. Thus, in order to discover novel biological processes associated with specific perturbations or disease [6–8] we need to consider data from complementary high-throughput datasets jointly.

Network modeling approaches allow us to overcome such limitations because they can combine multiple types of data without requiring prior pathway information. These approaches can either use user-generated or publicly available data, such as protein-protein interactions and epigenetic data, to find either direct or indirect (i.e. via unobserved molecules) connections between experimental hits. Critical to these approaches are protein-protein interaction databases, which collate data from multiple experimental platforms and cell types to provide networks of experimentally detected interactions [9–12]. In addition, recently generated protein-metabolite and protein-small molecule interaction networks, including HMDB [13], DrugBank [14] and STITCH [15], allow for richer assessment of molecular interaction types that occur in cellular systems. These collections of physical molecular interactions, or interactomes, enable researchers to apply network modeling approaches to a wide variety of data.

Measurements of transcriptional changes in response to perturbation or disease are a commonly generated omic data type. However, the proper approach for including transcriptional measurements in networks requires some thought. Since these data do not directly measure protein abundance or activity, it is misleading to map them directly to their corresponding proteins in the interactome. Instead, Omics Integrator combines such transcriptional data with epigenetic data to identify putative changes in the activity of DNA binding proteins that influence transcriptional changes [16].

**Fig 1:**
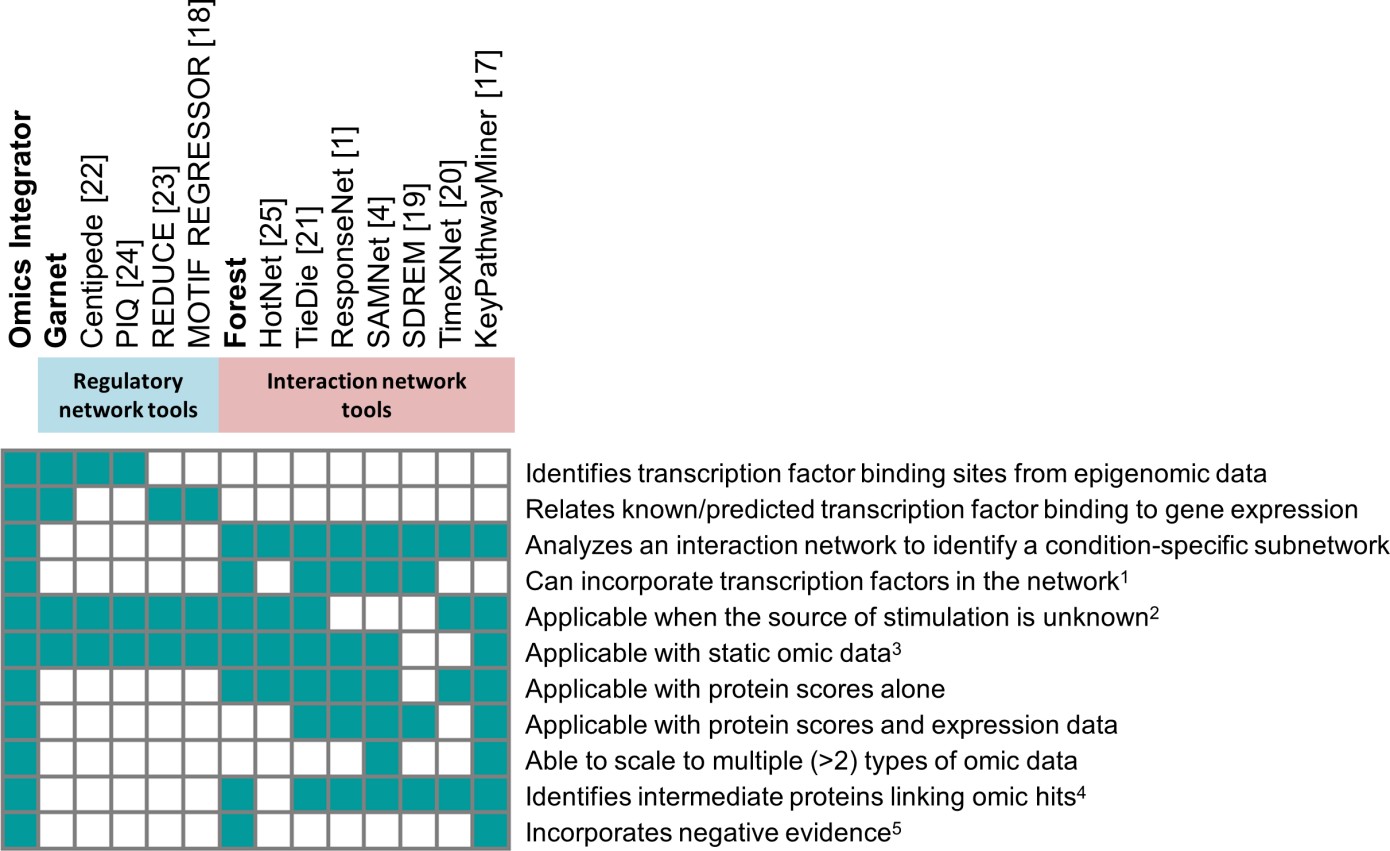
Summary of features differentiating Omics Integrator from existing tools and which features are available when Garnet and Forest are used individually. ^1^ Some network algorithms model TFs by including protein-DNA interactions in the network or generating TF scores for the protein nodes. ^2^ Some network algorithms optimize the transmission of information from source nodes to target nodes and require the sources to be identified in advance. ^3^ Time series analysis algorithms require omic data from three or more time points. ^4^ Intermediate proteins, like the Steiner nodes predicted by Forest, are not assigned condition-specific scores but are important for connecting other scored nodes in the subnetwork. ^5^ Negative evidence discourages network algorithms from selecting particular nodes due to prior knowledge or a bias, such as node degree.

This wealth of interaction data gives rise to new challenges. The published interactions between proteins, DNA, and small molecules comprise a network of millions of connections that is a ‘hairball,’ or a network that is too dense to interpret [5]. There are numerous individual tools that are now available to analyze these networks, each with different capabilities and intended application areas [1,4,17–25] (see **Fig 1**). Many network optimization methods that aim to reduce hairball interactomes to higher confidence subnetworks exist; however, many of these have limitations that inhibit their general applicability, such as requiring predefined source and target sets [1,4,20,26–29], which is not applicable in cases where omic data do not fit a ‘source-target’ framework. There are also methods that map mRNA expression datasets to protein interaction networks (MATISSE [30]), methods that identify transcription factor binding sites from epigenetic data (Centipede [22] and PIQ [24]), and methods that relate chromatin features and DNA-binding motifs to gene expression via multivariate/univariate regression (REDUCE [23] and MOTIF REGRESSOR [18]) or support vector regression [31]. These methods fall into two general classes: methods that attempt to reconstruct signaling pathways or interaction networks from data hits or methods that focus specifically on transcriptional regulatory networks. These two classes of tools are both essential to fully integrate diverse types of high-throughput data.

The tools currently available for omic data integration provide only a subset of the features provided by Omics Integrator (**Fig 1**). Furthermore, there is no existing algorithmic framework that enables the incorporation of weighted negative evidence. We define negative evidence in this context as any data or feature that supports potential exclusion of a species (protein, metabolite, etc.) from a network model solution (e.g. due to lack of expression in the system of interest, etc.). Supporting negative scores can help avoid misleading results in network analysis. KeyPathwayMiner [17] allows a list of negative nodes as input, but these are hard cut-offs. By contrast, Omics Integrator allows for weighted negative scores so the user can balance the prior evidence for excluding a node against the benefits of using it to connect nodes with positive scores.

In summary, we introduce Omics Integrator, a software package that fills a noticeable gap in omics data analysis by providing a unified framework for integrating transcriptomic data together with other omic data using interactome data. Although the individual components, Garnet and Forest, have similarities with existing tools, uniting expression analysis and network analysis in a single package makes it substantially easier to model multiple types of omic data. Omics Integrator expands upon and combines the prize-collecting Steiner forest (PCSF) algorithm [2,5,32] and methods similar to those implemented in previous network algorithms [4,33].

## Design and Implementation

**Fig 2:**
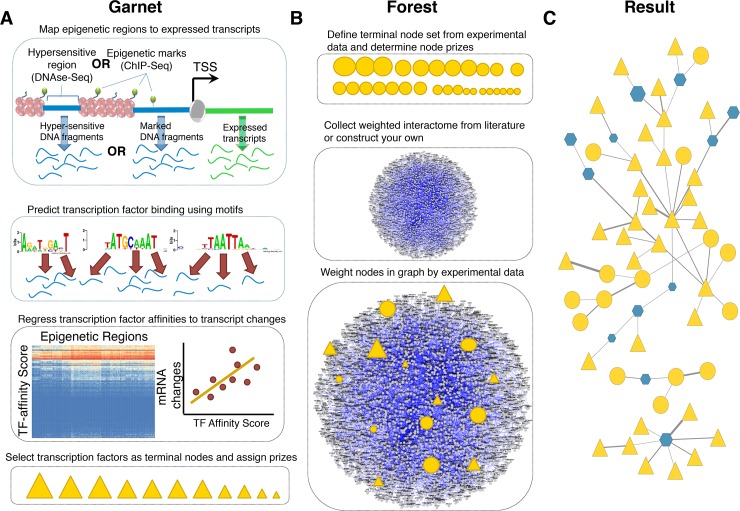
Summary of Omics Integrator **(A)** Garnet identifies transcription factors (triangles) associated with mRNA expression changes by incorporating epigenetic changes nearby expressed genes, scanning those regions for putative transcription factor binding sites and then regressing transcription factor affinity scores against gene expression changes. The result is a set of transcription factor candidates and the relative confidence that they are responsible for the observed expression changes. **(B)** Forest identifies a condition-specific functional sub-network from user data and a confidence-weighted interactome. The network can be composed of protein-protein, protein-metabolite or other interactions. The set of omic hits are composed of the TFs obtained from Garnet (triangles) merged with other types of hits such as differentially expressed proteins, significantly phosphorylated proteins, metabolites, etc. (circles). **(C)** Finally, the confidence-weighted interactome is integrated with the ‘omic’ hits using the prize-collecting Steiner forest algorithm, where the data is either connected directly or via intermediate nodes, called ‘Steiner nodes’.

The Omics Integrator package (**Fig 2**) consists of two distinct tools: Garnet and Forest. These tools work together to enable the integration of data derived from measurements of mRNA, proteins, genetic perturbations or metabolites.

Garnet takes chromatin accessibility data (e.g. DNase-Seq, histone modification ChIP-Seq), either generated by the user or acquired from public repositories (e.g. the ENCODE consortium [34], the NIH Roadmap Epigenomics Mapping Consortium [35]), and identifies a set of transcriptional regulators that potentially explain observed gene expression levels or changes between conditions in an experiment of interest (**Fig 2A**). From the chromatin accessibility data, Garnet scans regions proximal to transcribed genes for transcription factor binding sites. Binding sites are inferred from sequence matches in the underlying DNA to a clustered set [36] of DNA-binding motifs. Garnet then uses a pseudo-thermodynamic metric [37] to compute a transcription factor affinity (TFA) score in these regions and maps these scores to genes within a fixed window (e.g. 2kb). Garnet estimates transcription factor activity by performing univariate linear regression of the TFA scores against corresponding mRNA expression measurements. Significant regression coefficients indicate candidate transcriptional regulators that can then be used as input to the Forest program or analyzed independently.

The Forest tool identifies a parsimonious interaction network connecting a subset of user-defined omic data hits (**Fig 2B**). These hits can be the transcription factors selected by Garnet and/or any other type of biological data (e.g. siRNA hits, phosphoproteomic changes, metabolites, etc.). Specifically, Forest solves the prize-collecting Steiner forest problem [5] that takes into account the importance assigned to the omic hits (e.g. by significance level or fold-change between conditions) as well as the probability that each reported interaction is real. Each omic hit and/or Garnet-selected transcription factor is given a positive ‘prize’, reflecting the confidence in the reliability of the underlying data (e.g. a set of differentially expressed proteins). These user-defined hits are referred to as terminals. When the algorithm includes a terminal in the network, it is rewarded with the prize assigned to that hit, but also has to pay costs for the interactions used to link the data to the rest of the network. By seeking to maximize the collected prizes and minimize the edge costs, the algorithm uncovers a high-confidence set of physical interactions that explain how the omic hits are related. Because the algorithm is not forced to include all omic hits, it can remove those that are poorly connected and give rise to the ‘hairball’. However, Forest can at the same time select additional, interactome-derived nodes called ‘Steiner nodes’ when necessary. Steiner nodes are likely relevant to the biological response in question, but may be missed by the high-throughput assays. Forest generates output files that can be easily viewed using the network visualization software package Cytoscape [38].

Forest includes numerous features to efficiently generate biological networks, while avoiding common pitfalls often encountered when implementing network-based algorithms. For example, many network methods are inherently biased towards using nodes that have been studied more extensively and have more reported interactions [39–41]. Forest can penalize these highly-connected nodes, termed ‘hub’ nodes here, by assigning to them a prize with a negative value. Without the negative prizes, hub proteins are often selected even when they are not biologically relevant or interesting. With negative prizes, they only appear in the network when other data strongly implicate them. Additionally, Forest includes randomization strategies to ensure that the resulting networks are robust to noise in the biological data. We include in our software package a straightforward example in which phosphoproteomic measurements are integrated with changes in mRNA expression [42] using epigenetic data from a related cell line from ENCODE [34,43]. We also include examples in which Garnet and Forest can be run independently, described in more detail on our website: http://fraenkel.mit.edu/omicsintegrator.

### Mapping gene expression changes to proteins

The Garnet algorithm reveals candidate transcriptional regulators that likely influence gene expression levels or changes. We prefer to not use mRNA measurements as proxies for changes in protein levels or activities as the relationship between mRNA and protein levels is complex [44–46]. In addition, changes in protein concentrations are not reliable evidence for changes in the activities of pathways, which are often post-translationally regulated. Once Garnet identifies transcription factors that give rise to the observed mRNA changes, the transcription factors can then be used as input to Forest.

Garnet is a core part of omics integration, enabling gene expression changes to be mapped to transcription factors in the interactome that can then be then analyzed alongside other data [47]. Garnet builds on the rich epigenetic datasets that have been collected through consortia such as ENCODE by using data from histone modifications or open chromatin experiments to restrict the search space for motif matches to areas more likely to be bound by some regulator. This strategy has been used by this lab [48] and others [49] to reduce spurious motif matches and thus to improve the accuracy of transcription factor binding prediction [22]. Although this type of inference cannot eliminate uncertainty about the binding of a TF to a specific site or gene, the cumulative evidence is predictive of the activity of the TF in a transcriptional response. As Omics Integrator solves the prize-collecting variant of the Steiner problem, it can exclude false positives that are difficult to connect to upstream signaling. Omics Integrator also provides options to filter the candidate TFs from Garnet, including checking whether or not the TF is expressed in the tissue or cell type of interest.

Garnet consists of two steps: (1) computationally predicting transcription factor-DNA interactions from epigenetic data and a set of DNA binding motifs and (2) estimating regulator activities by correlating these predicted transcription factor-DNA interactions with mRNA expression changes in genes neighboring the predicted binding sites.

#### Epigenetic data processing

Garnet first finds genomic regions that likely harbor transcriptional regulatory proteins by searching enriched open chromatin regions for matches to DNA binding protein motifs. These regions are typically identified using epigenetic data either within a similar cell type or from conserved regulatory regions across cell or tissue types [50]. Garnet first searches a list of chromatin regions, provided by the user or included with Omics Integrator, and assigns the regions to genes with open chromatin sites within a user-defined distance threshold (e.g. 2000 base pairs). Garnet then scans these regions to determine the likelihood that a transcription factor will bind a region using a set of position weight matrices (PWMs).

#### Transcription factor binding prediction

Each region of DNA associated with an epigenetic signal is given a score representing the probability of binding for each motif matrix using a statistical-mechanics framework, where the number of possible binding sites and their PWM scores in each sequence are combined to create a single transcription factor binding probability for each region. The Boltzmann weighted partition function below is used for each motif and chromatin region and has been described in this context previously [37]. The equation to define transcription factor affinity is:

$${TFA}_{j}=\frac{\sum_{i}e^{w_{j}∙m_{i}}}{\beta_{j}+\sum_{i}e^{w_{j}∙m_{i}}}$$ (1)

*TFA_j_* estimates the probability of binding for motif *j* using all scoring windows *i* in the region. *w*_*j*_ estimates the probability that the motif score is not a false positive, *m*_*i*_ represents the PWM log-likelihood score at the *i*^th^ window in the region, and *β_j_* estimates the probability that motif score is a false positive. In practice we use *w*_*j*_ and *β*_*j*_ as tuning parameters based on the TRANSFAC MATCH minSUM and minFP score thresholds for each motif. For genes with multiple epigenetic regions within the associated window, Garnet chooses the highest *TFA_j_* value for each motif *j* across all regions. The result is a matrix representing the affinity score for each gene and each transcription factor binding motif.

#### Transcription factor selection

Garnet uses linear regression to identify motifs mapped to transcription factors with the strongest relationships to the expression data. This approach is similar to that in previous work [37] and assumes that the better the match of a sequence to a motif (summed over an epigenetic region), the stronger the binding and the greater the effect the regulator has on transcription. We apply least-squares regression to relate the *TFA* scores described above to mRNA expression changes for a particular condition of interest. Significance is assessed by testing the null hypothesis that the slope of the regression line is 0. Transcription factors with motifs exhibiting statistically significant regression coefficients (p-value<0.05 or any desired threshold) are given a weight of–log(p-value).

### Network modeling

#### Selection of the terminal node set and assignment of prizes

To map experimental data to an interactome of interest (**Fig 2B**), the user must first identify the most biologically significant hits from each dataset and define them as terminal nodes in the network. Terminal nodes are any entities represented by nodes in the network that the user would like the algorithm to analyze in a larger biological context. Typically, these are molecules that change significantly under a treatment relative to an appropriate control. Prizes, denoted p(v), where v is a vertex (node) in the interactome graph, are assigned to the terminal nodes by the user. These prizes can be, for example, the log fold change of proteins in an experiment or negative log of the significance level describing changes between conditions (p-value or q-value). If there is no quantitative information and only a set of terminal nodes is available, users can assign uniform prizes to each terminal.

As already noted, Forest can use node prizes to incorporate negative evidence about the relevance of a node. A priori, it is impossible to know if such a protein has a high degree because it is truly involved in many interactions or because it has been studied more extensively than other proteins because it is highly-conserved, essential, or highly-expressed [39–41]. To avoid the potential bias introduced by these hub nodes, we created a generalized prize function that assigns negative weights to nodes based on the number of connections they have in the interactome. As a result, hubs are less likely to be selected but can still be used when the data strongly support their inclusion. The function for this negative weighting is:

$$p^{\prime }(v)=\beta ∙p(v)-\mu ∙degree(v)$$ (2)

where *degree(v)* is the number of connections of node v in the interactome. The *β* and *μ* parameters are scaling factors to adjust the effect of terminal nodes and hub nodes in the final network, respectively. When *μ* is set to 0, the hub correction is disabled (default behavior). Increasing *μ* attenuates the hub dominance in the optimal solution. Increasing *β* promotes more terminal nodes to be included in the optimal solution (Eq 2).

In addition to reducing the influence of hub proteins, negative prizes could be used to reduce the influence of molecules that are poorly expressed in a particular tissue or condition. Similarly, negative prizes could be used to exclude molecules that have been experimentally determined to not be relevant to the process under study. Users can take advantage of this feature by simply adding negative values to the original prize file.

#### Confidence-weighted interactome and edge costs

Calculating the probability p(e) that an edge e between two proteins reflects a real interaction allows us to avoid false positive edges, which are assumed to be less reproducible and therefore less confident. Forest takes as input a set of edge weights (p(e)) and converts them to costs using the scoring function c(e) = 1 –p(e). Several approaches have been described for deriving these probabilities or other confidence scores [1,10,26,51,52].

### Forest problem formulation

The input to Forest is a directed, partially directed, or undirected network *G(V*, *E*, *c(e)*, *p’(v))* of node set *V* and edge set *E*, where the function *p’(v)* assigns a prize to each node *v ∈ V* and the function *c(e) > 0* assigns a cost to each edge *e ∈ E*. The aim is to find a forest *F(V*_*F*_,*E*_*F*_*)* that minimizes the objective function:

$$f^{\prime }(F)=\sum_{v\notin V_{F}}p^{\prime }(v)+\sum_{e\in E_{F}}c(e)+\omega ∙\kappa$$ (3)

where *p’(v)* is as defined in Eq 2, *κ* is the number of trees in the forest, and *ω* is a tuning parameter whose purpose is explained below. Here, an artificial node, or ‘dummy’ node, *v*_*0*_ is introduced to the initial network and connected to a subset of nodes *N* (using the --dummyMode option detailed below). The nodes in *N* are a subset of all the nodes in the interactome *V* and are assigned a uniform edge cost *ω* to the dummy node. Forest constrains *F* to be a tree–a connected graph without cycles–that is rooted at *v*_*0*_. The optimization problem is solved with the msgsteiner message-passing algorithm to identify a tree subnetwork [53]. Once the network optimization problem has been solved, the root node (*v*_*0*_) and all its edges are removed, providing a final forest network that is a collection of one or more sub-trees. These sub-trees conceptually represent parallel biological pathways. Given that the resulting solution is dependent on the particular values of β, ω, and μ, we suggest that the user run the algorithm with different settings to select an optimal solution. We recommend choosing optimal parameters based on two criteria. First, the parameters should maximize fraction of terminal nodes included in the network that are also robust to noise; this robustness can be determined by permutations derived from the --noisyEdges flag. Among the parameter settings that yield a similar fraction of robust terminals, we prefer larger networks. It is often also helpful to check that the selected hidden nodes are enriched for biologically-relevant categories using known pathways or gene sets (e.g. from MSigDB [54] or Gene Ontology [55]).

Up to six PCSF parameters are supplied to Forest in a configuration file. The minimum required parameters are *ω*, *β* and *D*. The parameters *ω* (controlling the number of trees; Eq 3) and *β* (controlling the trade-off between including more terminals and using less reliable edges; Eq 2) are as described above. *D* (the depth parameter) controls the maximum path-length from *v*_*0*_ to terminal nodes. Suggested values for D range between 5 and 10. The optional parameters are μ, *g*, and *garnetBeta*. The parameter μ controls the degree-based negative prizes (Eq 2); if not provided, μ is assumed to be zero. The reinforcement parameter, *g*, affects the convergence of the solution (smaller values produce solutions closer to the optimum, but increase run time) and is set to 1e-3 by default. The *garnetBeta* parameter is used to scale the Garnet output prizes relative to the provided protein prizes. The default for *garnetBeta* is 0.01.

### Additional algorithm features

Forest converges on a single, optimal solution. However, it can be useful to perform perturbation analyses to determine the robustness of this network and how it relates to suboptimal solutions. Forest provides three different perturbation strategies. To ascribe confidence in the selected hidden nodes, the user can use the --noisyEdges flag to assign noise to the edges of the interaction network. Hidden nodes that appear often in networks run with noisy edge weights are likely more robust than those that only appear in the optimal solution or a small number of noisy runs. A user can also use --shufflePrizes to identify those hidden nodes that are robust to noise in the prize data. Lastly, the user can also assess specificity of hidden nodes using the --randomTerminals flag that runs the optimization with a random selection of terminals (preserving the degree distribution of the original terminal set). Hidden nodes that occur less frequently in forests run with random prizes are likely to be more specific to the user’s problem of interest and therefore more biologically meaningful. The results of these perturbations can either be used to weight nodes and edges in the original network using the ‘fraction of optimal networks included’ attribute or viewed together using the merged network produced by the algorithm.

As previously mentioned, Forest incorporates a dummy node into the graph when solving the optimization problem. When this dummy node is removed after the optimization, the solution is divided into disjoint subnetworks. The --dummyMode option tells the algorithm which nodes in the interactome should be connected initially to the dummy node. The default option (--dummyMode terminal) connects the dummy node to all of the input terminals, guaranteeing that each sub-network in the optimal solution is rooted by a terminal node. The option --dummyMode filename allows the user to explicitly specify which nodes to connect to the dummy node. For example, in our previous work, we used this option to identify signaling pathways originating at cell-surface receptors [5]. There are additional values for the --dummyMode option, recommended for advanced users, as described in **Table 1**.

**Table 1:** Description of the parameters used in the Forest.py script.

-p PRIZEFILE, --prize=PRIZEFILE	Path to the text file containing the prizes	Path to a tab-delimited plain text file with lines “ProteinName PrizeValue”
-e EDGEFILE, --edge=EDGEFILE	Path to the text file containing the interactome edges	Path to a tab-delimited plain text file with 3 or 4 columns: “ProteinA ProteinB Weight(between 0 and 1) Directionality(U or D, optional)
-c CONFFILE, --conf=CONFFILE	Path to the text file containing the parameters. Default = “./conf.txt”	Path to a tab-delimited plain text file with lines “ParameterName = ParameterValue”. Must contain values for w, b, D. Optional parameters mu, garnetBeta, g may also be included
-d DUMMYMODE, --dummyMode=DUMMYMODE	Tells the program which nodes in the interactomes to connect to the dummy root node. Default = “terminals”	Either a file name (containing a list of nodes), “terminals” (connect to all prize nodes), “others” (connect to nodes with no prize), or “all” (connect to all nodes)
--garnet=GARNETOUTPUT	Tells the program that it will also use the Garnet output for network modeling. The prizes will be scaled by the garnetBeta parameter you provide in the conf file, default 0.01	Full path + filename of the Garnet output file
--musquared	Flag to add negative prizes to hub nodes proportional to their degree^2^, rather than degree. Use to penalize hub nodes more intensely. Must specify a positive mu in conf file.	
--msgpath=MSGPATH	Path to the msgsteiner executable, including the executable name. Default = “./msgsteiner”	Path where the msgsteiner executable is located
--outpath=OUTPUTPATH	Path to the directory which will hold the output file. Default = this directory	Path
--outlabel=OUTPUTLABEL	A string to put at the beginning of the names of the files output by this program. Default = “result”	String
--cyto30	Use this flag if you want the output files to be compatible with Cytoscape v3.0 (this is the default)	
--cyto28	Use this flag if you want the output files to be compatible with Cytoscape v2.8	
--noisyEdges=NOISENUM	Specifies how many times you would like to add noise to the given edge values and re-run the algorithm. Results of these runs will be merged together and written in files with the word “_noisyEdges_” added to their names. Default = 0	Integer
--shuffledPrizes=SHUFFLENUM	Specifies how many times you would like to shuffle the given prizes around the terminals and re-run the algorithm. Results of these runs will be merged together and written in files with the word “_shuffledPrizes_” added to their names. Default = 0	Integer
--randomTerminals=TERMNUM	Specifies how many times you would like to apply the given prizes to random nodes in the interactome (with a similar degree distribution) and re-run the algorithm. Results of these runs will be merged together and written in files with the word “_randomTerminals_” added to their names. Default = 0	Integer
--knockout=KNOCKOUT	Specifies protein(s) you would like to “knock out” of the interactome to simulate a knock-out experiment	The name(s) of the protein(s), i.e. TP53 or TP53 EGFR
-s SEED, --seed=SEED	A seed for the pseudo-random number generators. If you want to reproduce exact results, supply the same seed. Default = None	Integer

Forest can also automatically perform *in silico* knock-out experiments, i.e., identifying new solutions when a specific node removed from the interactome. These can be valuable for determining the robustness of the solution and the significance of individual nodes [5]. To use this option, the user specifies --knockout and provides node names specifying node(s) the user would like to ‘knock out’, i.e. TP53 or TP53 EGFR.

**Fig 3:**
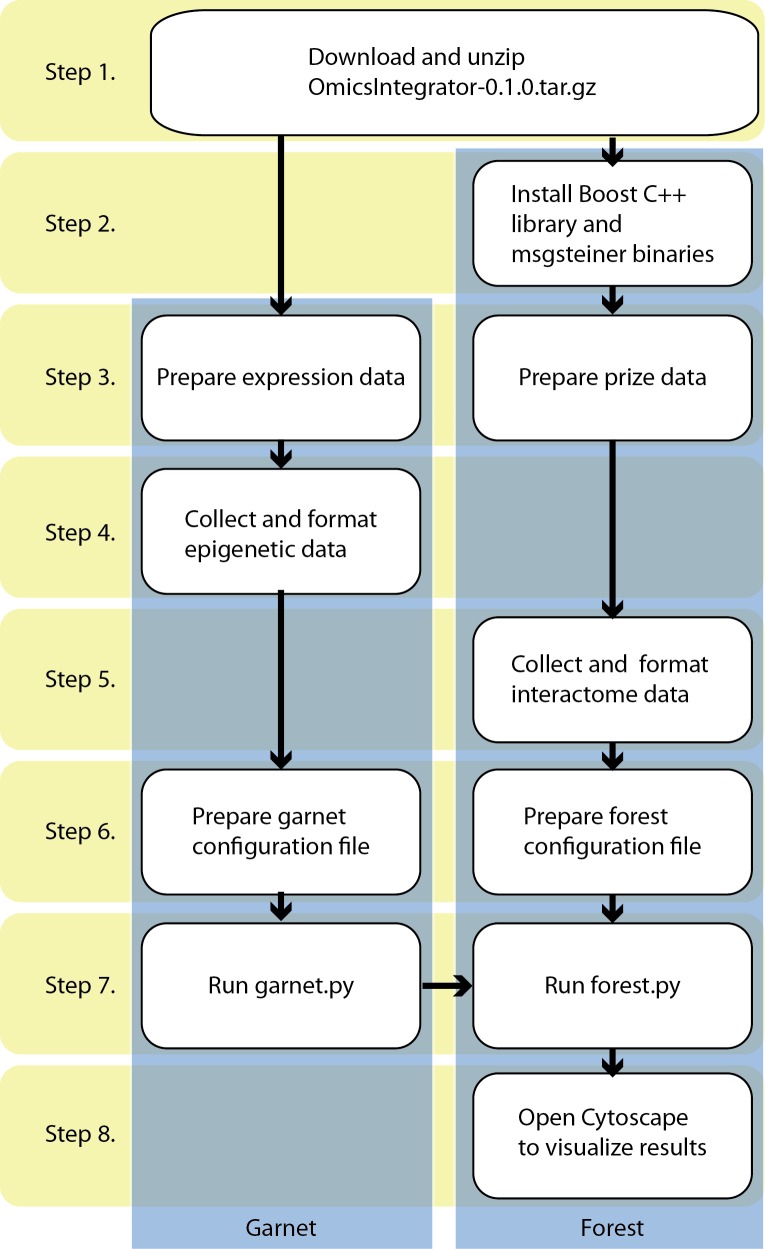
The flowchart of the software. Step 1 requires downloading and unzipping the scripts and data files. Step 2 consists of the installation of the necessary tools to run Omics Integrator. Step 3 describes how to prepare input files. Step 4 and 5 are designed for data collection and formatting for Garnet and Forest modules, respectively. At Step 6, configuration files are prepared where parameters are defined for Garnet and Forest separately. Garnet and Forest scripts are run at Step 7. If the initial data contains transcriptional data, then Garnet must be run before Forest. Otherwise Forest can be run independently. Detailed instructions of these steps are in the ‘Procedure’ section of the S1 Text.

There are other Forest options in addition to those explained here. The full list is provided in **Table 1**. The step-by-step procedure to run Omics Integrator and troubleshooting guidance are provided in the **Supplementary Material**. A flowchart showing how to run Omics Integrator is depicted in **Fig 3**.

## Results

### Omic data integration and network reconstruction in lung carcinoma

To showcase the utility of Omics Integrator, we analyzed several types of omic data from lung carcinoma cells. We collected previously published data [42] from H358 cells, a model of lung cancer, that were stimulated with TGF-β. Measured gene expression changes were used as input into Garnet together with DNase I hypersensitive regions from A549 cells, a related lung carcinoma-derived cell line. The resulting transcription factors identified by Garnet were used, together with phosphoproteomic expression changes from the same experimental conditions, as input into Forest. Forest then found a collection of edges from the protein interaction network that connected the two classes of nodes with TGF-β receptors.

**Fig 4:**
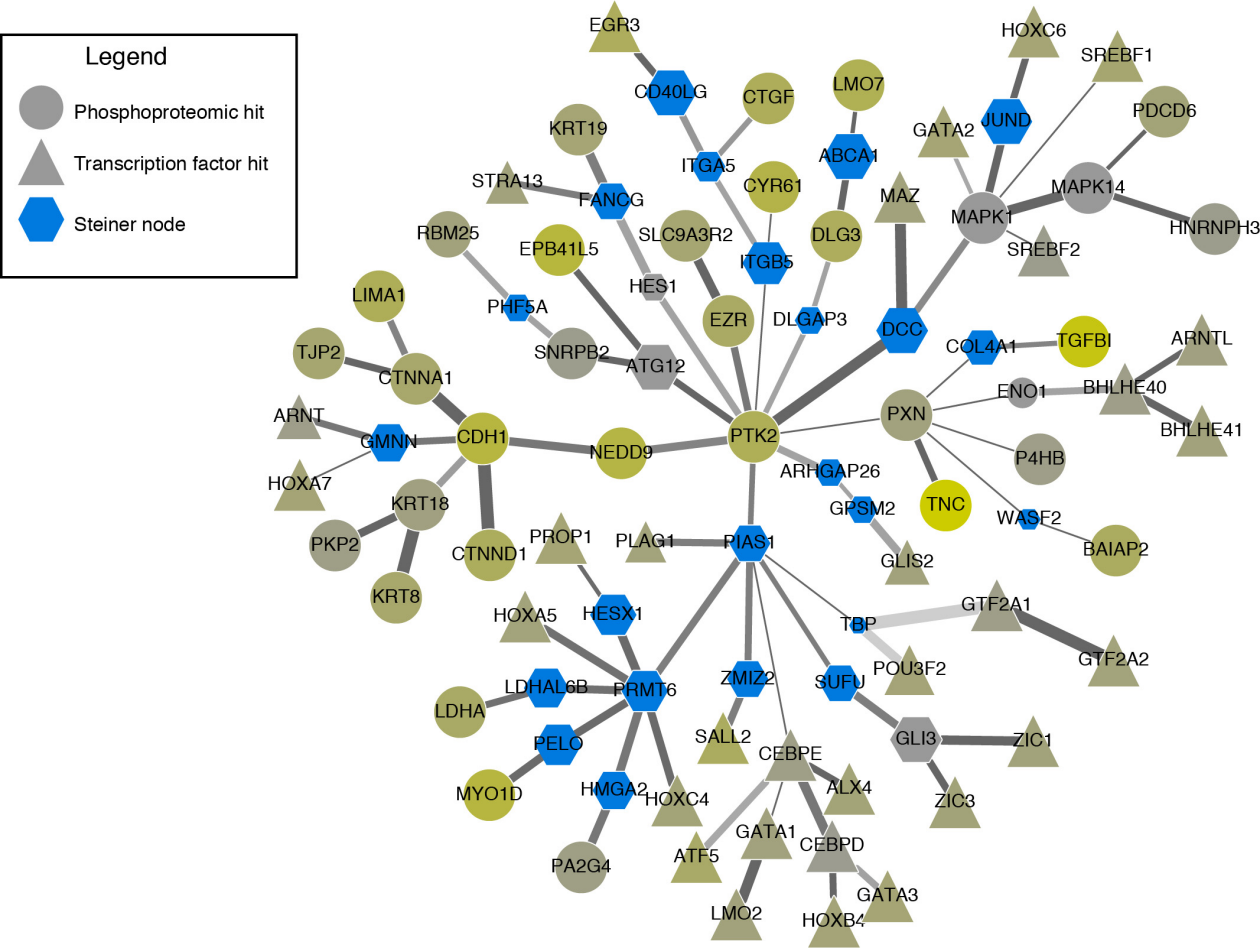
Anticipated results: Network reconstructed from changes in phosphoproteomic measurements (circles) and gene expression measurements (triangles) in lung cancer cell lines stimulated with Tgf-β. Blue hexagons represent ‘Steiner nodes’ that were not measured as changing in the original experimental measurements but identified through network reconstruction. Nodes that are not blue were measured in the phosphoproteomic data, with color indicating the degree of change in phosphoproteomic measurements: grey indicates no change and yellow indicates a large amount of change. Network robustness was measured by adding noise to the edges using the --noisyEdges flag. The shade of the edge is correlated with the number of times the edge was selected over all perturbations, and the size of a node represents number of times the node was selected. The width of the edge represents the weight assigned to the interaction in the original interactome.

The resulting network, depicted in **Fig 4**, showcases the ability of the forest algorithm to connect known targets (derived from phosphoproteomic and expression data) using the protein-protein interaction network as well as identify hidden ‘Steiner’ nodes (hexagons) that interact with Garnet-identified transcription factors (triangles) and proteins that exhibit phosphorylation changes (circles). Included among the more robust Steiner nodes (node size correlates with robustness to perturbation) are proteins that have been linked to EMT signaling in cancer, such as PIAS1, a SUMO E3 ligase that is repressed by TGF-β to prevent EMT suppression [56], and COL4A1, which has also been linked to TGF-β stimulation [57]. SUFU and GLI3 have been linked to Hedgehog signaling [58], another pathway in cancer [59], suggesting a putative explanation for the link between TGF-β and Hedgehog signaling. Additional Steiner nodes present in our network, including ABCA1 and ATG12, to the best of our knowledge have not been studied in this context and may point to novel aspects of the TGF-β signaling pathway.

To generate the network illustrated, users can run the test-tgfb-data.py script in the example/a549 directory of the OmicsIntegrator package. This script will run Garnet using the ENCODE-derived DNase I hypersensitive data from A549 cells with the gene expression data from related cells. This script then runs Forest using a scored version of the iRefIndex interactome (version 13) [9] provided with Omics Integrator to identify links between the Garnet transcription factors and the proteins phosphorylated upon TGF-β stimulation.

### Network modeling and in silico knock-out experiment in human primary Glioblastoma cells

**Fig 5:**
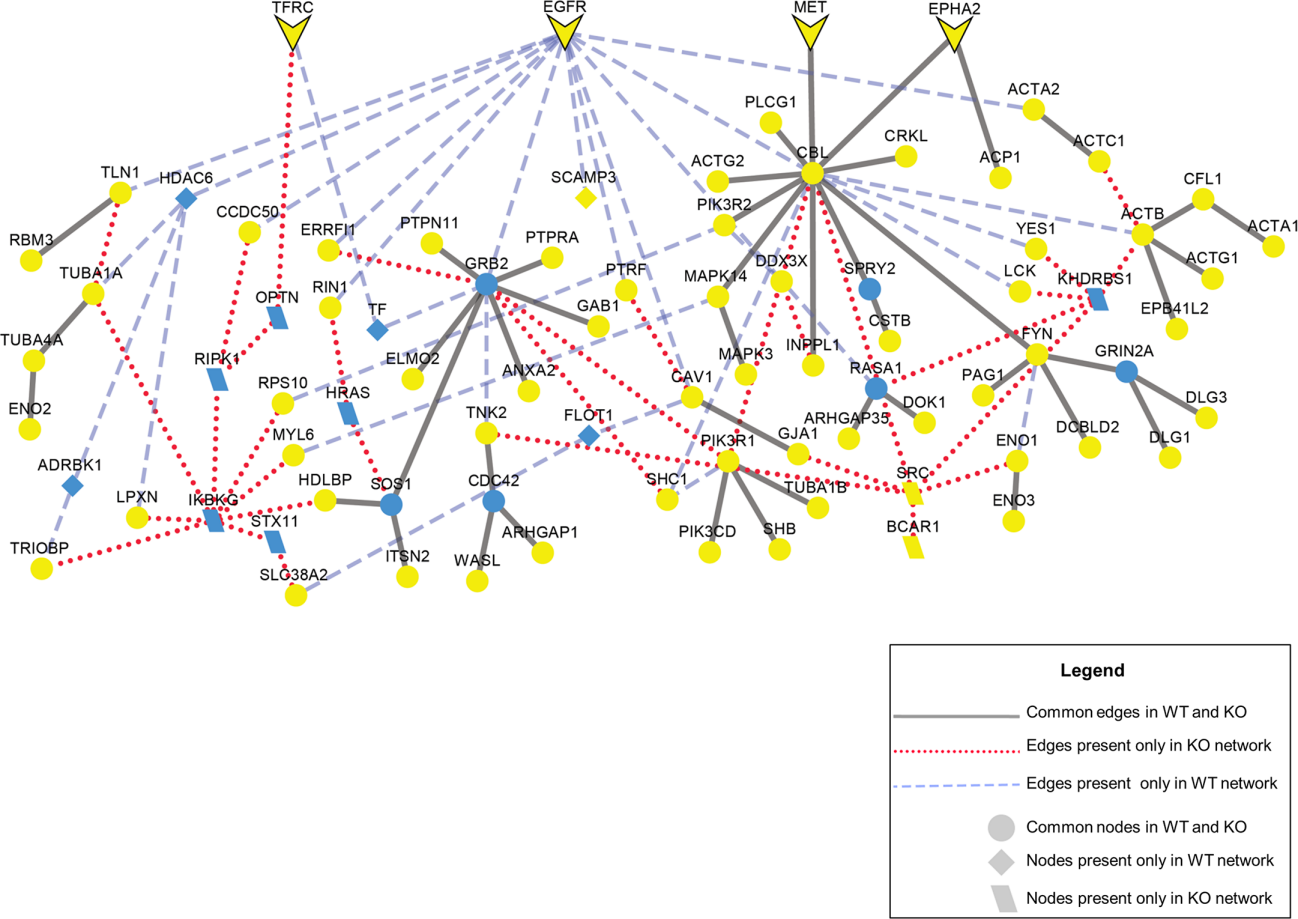
Anticipated results: In silico EGFR knock-out experiment in network modeling. Blue nodes represent ‘Steiner nodes’ that were not measured as changing in the original experiment but are identified through network reconstruction; yellow nodes represent ‘terminal nodes’ that are the phosphoproteomic hits. The original network and the network with EGFR knock-out have been merged to clearly show the common and different nodes and edges in the two conditions. Common edges in two conditions are black lines, edges only present in EGFR knock-out condition are red dotted lines and edges only present in the wild-type condition are blue dashed lines. Cell surface receptors are arrow-shaped. The parameters are μ = 0.002, ω = 2, β = 150, and D = 10.

Forest also has the ability to perform *in silico* knock-outs of specific nodes to model loss or knock-out of these species from a system. Such knockouts can be useful to examine signaling that can occur after a receptor has been inactivated through mutation or pharmacological treatment. For example, the epidermal growth factor receptor (EGFR) is the target of tyrosine kinase inhibitors including erlotinib and gefitinib. To determine the alternate signaling pathways that could function in the presence of such inhibitors, we use the Forest algorithm to construct a network from phosphoproteomic data measured in U87 cell lines, a model of glioblastoma tumors which has been published previously [60]. We compared this network, called the wild type (WT) network, with a second network built from the same data, but without EGFR included in the interactome (knock-out or KO network). A comparison of the WT and KO networks is depicted in Fig 5. When EGFR is removed from the interactome, the blue dashed edges are removed in the final network, but many key signaling nodes, such as GRB2, CBL and PIK3R1, remain. Also, several cell surface receptors, such as MET, TFRC, EPHA2 are robust to EGFR removal. The network suggests that these receptors could continue signaling to the same downstream targets of EGFR. Indeed, crosstalk between MET and EGFR has been previously identified [61]. These results suggest the presence of alternate pathways that could contribute to the failure of some glioblastoma tumors to respond to treatment.

### Negative prize weighting reduces bias in interaction networks

**Fig 6:**
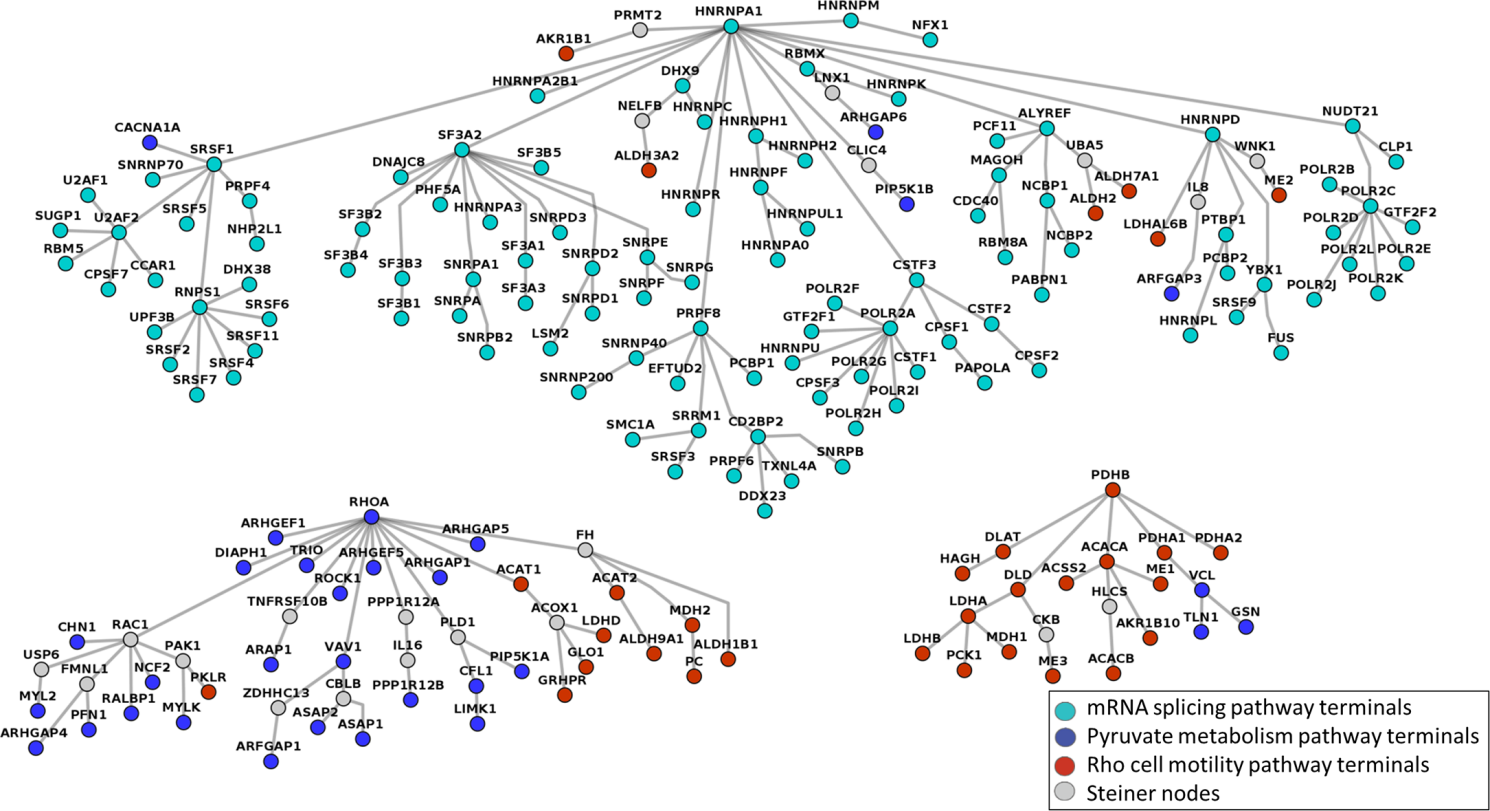
The final PCSF reconstructed from the terminal set formed by the members of mRNA splicing pathway, pyruvate metabolism pathway, and Rho cell motility pathway in ConsensusPathDB. Each node is colored according to the pathway to which it belongs, and Steiner nodes are colored gray. The parameters are μ = 0.009, ω = 3, β = 5, and D = 5.

Negative prizes can improve the accuracy of Forest’s reconstructed networks. We tested the efficacy of adding negative prizes by testing the ability of Forest to reconstruct annotated pathway data from ConsensusPathDB as separate trees [62]. We collected proteins from three pathways in ConsensusPathDB: mRNA splicing, pyruvate metabolism and Rho cell motility. In theory, we would expect these proteins to form three independent trees using the Forest algorithm because they are biologically distinct processes. Without negative prizes, however, Forest assembles all nodes into a single tree (see Fig A in S1 Text). When we penalize high-degree nodes using negative prizes using the μ parameter, the nodes from each pathway form distinct subtrees, illustrated in **Fig 6**. In addition to recapitulating annotated pathways, nodes in each subtree are enriched for distinct GO processes and shown in Fig B in S1 Text.

## Availability and Future Directions

Omics Integrator is an open-source project licensed under the Creative Commons Attribution-NonCommercial 4.0 International Public License. It is available for download at http://fraenkel.mit.edu/omicsintegrator. The install instructions will ensure that all required Python libraries are installed. To solve the prize-collecting Steiner forest problem, Forest uses the message-passing algorithm msgsteiner, which requires a C++ compiler and the Boost library (www.boost.org). The msgsteiner source code can be downloaded from http://areeweb.polito.it/ricerca/cmp/code/bpsteiner. For installation, follow the guidelines in the downloaded files. Development of Omics Integrator is ongoing through our GitHub site (https://github.com/fraenkel-lab/OmicsIntegrator), which provides a framework for collaborations across institutions.

Future directions include adding support for more types of interactions (such as protein-RNA interactions), multiple sets of prizes derived from patient data [32], and additional operating systems. We also plan to improve the parameter selection process and Garnet execution time.

## Supporting Information

S1 Text Supplementary material. Detailed procedure to run Omics Integrator Software and interpret the results.
(DOCX)
