# Supplementary material for: Network-Based Interpretation of Diverse High-Throughput Datasets through the Omics Integrator Software Package
Source: PLoS Comput Biol. 2016 Apr 20;12(4):e1004879. doi: 10.1371/journal.pcbi.1004879 (PMC4838263; doi:10.1371/journal.pcbi.1004879)
Supplement: S1 Text — Detailed procedure to run Omics Integrator Software and interpret the results. (DOCX) [file pcbi.1004879.s001.docx]

**S1 Text.**

**
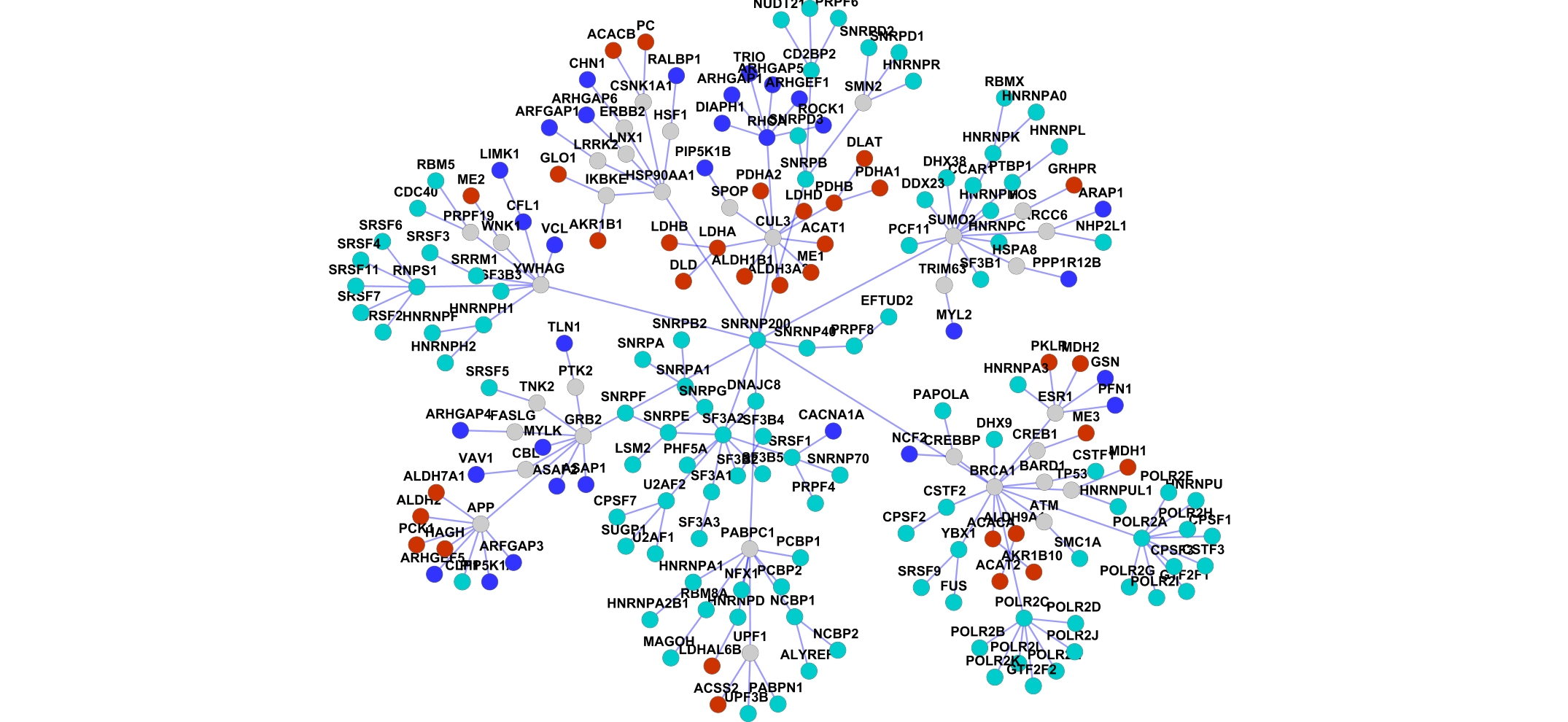
**

**Figure A.** The PCSF reconstructed from the terminal set formed by the members of mRNA splicing pathway, pyruvate metabolism pathway and Rho cell motility pathway in ConsensusPathDB without negative prizes (μ=0.0, ω=3, β=5, D=5). The same coloring scheme with Figure 5 has been used. Note that, compared with Figure 5, the processes in this network are much less separated due to the effect of hub nodes.

**Figure B.** Heatmap depicting GO terms enriched in the individual subtrees identified in Figure 5. Each individual GO term, labeled along the rows, is over-represented (enrichment p-value is represented by color of square in heatmap) in only one subtree. The lack of overlap between terms for the various subtrees illustrates Forest’s ability to identify biologically distinct trees.

**
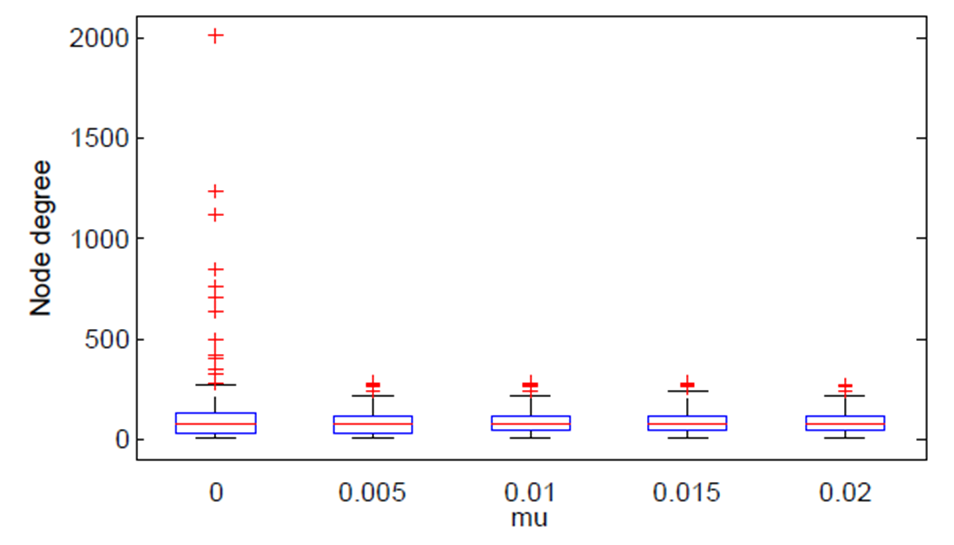
**

**Figure C.** The degree distribution of nodes in the final PCSF calculated from the overall interactome.

**Supplemental Methods.**

**1. Equipment and Setup.** A computer running Unix/Linux or Mac OS and having an internet connection. To run the provided scripts, Python 2.6 or 2.7 is required. The Python scripts can be downloaded from our website at <http://fraenkel.mit.edu/omicsintegrator> and the install instructions will ensure that all required Python libraries are installed.

1. **Download and install.**

To install the software, download the package from the software website (<http://fraenkel.mit.edu/omicsintegrator> ) and unpack the downloaded file. You can then open the directory and use pip to ensure that you have the required Python libraries.

$ tar –xvzf OmicsIntegrator-0.1.0.tar.gz

$ cd OmicsIntegrator-0.1.0

$ pip install –r requirements.txt

We recommend that users without an existing Python environment install Anaconda (<https://www.continuum.io/downloads>) to obtain Python 2.7 and the following required packages:

- numpy: <http://www.numpy.org/>
- scipy: <http://www.scipy.org/>
- matplotlib: <http://matplotlib.org/>
- Networkx: [http://networkx.github.io](http://networkx.github.io/)

Cytoscape is needed to visualize the resulting networks [[1](#_ENREF_1)]. Follow the instructions at <http://www.cytoscape.org/download.html> to download and install Cytoscape. Omics Integrator supports Cytoscape versions 2.8 and 3.0.

1. **Download Boost C++ library (if needed)**

The Boost C++ library is also required for the installation ([www.boost.org](http://www.boost.org)). Boost is pre-installed on many Linux distributions. If the operating system does not include Boost, follow the Boost getting started guide for instructions on how to download the library and extract files from the archive. To use the Homebrew package manager for Mac simply type brew install boost to install the library.

1. **Download msgsteiner code**

We recommend installing the msgsteiner code in the OmicsIntegrator-0.1.0 directory. To test the installation, we recommend running a test example in which only the forest.py script is called:

$ cd OmicsIntegrator-0.1.0/example/a549

$ python test-tgfb-data.py –-forest-only –-msgpath=[PATH TO MSGSTEINER]

To solve the prize-collecting Steiner forest problem, Forest uses the message-passing algorithm msgsteiner. A C++ compiler is needed to install msgsteiner. The msgsteiner source code can be downloaded from <http://areeweb.polito.it/ricerca/cmp/code/bpsteiner>. For installation, follow the guidelines in the downloaded files.

**2. Procedure**

This section explains how to run Omics Integrator.

1. **Preparing the input data**

**Response data files for Garnet**

The user should submit to Garnet a list of genes that change significantly in the condition of interest. Submitting a complete list of expression data for all genes will lead to poor results. Transcriptional data is provided as input into garnet.py. Expression response data is a plain tab-delimited text file where the first column lists the name of the gene and the second column is the log-fold change of the gene expression. An example of response data is present below.

ABCA12 -1.85675

ACCS 1.38648

ACSBG1 -4.20762

ADAM19 -0.710875

**Prize files for Forest**

Any type of data at protein level (i.e. proteomic, phosphoproteomic, mutation, etc.) can be provided as prizes in the forest.py script. The prize file is a plain text, tab-delimited file composed of two columns with an optional header line. The first column contains the list of proteins and the second column contains the prize values, which are positively correlated with the significance of a protein in a treatment condition relative to a control condition. We recommend that prizes range from 0 to 1, though the beta parameter can be used to scale the prizes up or down as needed.

1. **Getting fasta sequences around the peak summits in the bed file.**

Interpretation of gene expression data by garnet.py is improved by condition-specific epigenetic data such as DNase-I hypersensitive data or histone marks. You can collect your own data and then use a peak-finding algorithm (i.e. MACS[[2](#_ENREF_2)], GPS[[3](#_ENREF_3),[4](#_ENREF_4)] etc.), use data from ENCODE, or use the data provided in the example directory. Once you have the data in BED format, you need to collect fasta-formated sequence data corresponding to those regions as follows:

1. Browse to the Galaxy web tool (<https://usegalaxy.org>)
2. From the left panel, upload the bed file. (Get data → Upload File)
   1. Select the File Format as bed.
   2. Select the correct genome. In the anticipated results Human Feb.2009 has been selected.
   3. Wait until the file is uploaded.
3. From the left panel, select **Fetch Sequences** → **Extract Genomic DNA**; then, click ‘**Execute**’
4. After step c was completed, download the resulting .fasta file from the Galaxy server to be used for the coming parts of the software.
5. **Prepare the input interactome.**

We provide a weighted human interactome from iRefIndex (v13) [[5](#_ENREF_5)] with MI-score[[6](#_ENREF_6)] edge weights as part of Omics Integrator: data/iref_mitab_miscore_2013_08_12_interactome.txt. If you choose to create your own network, the interactome should be provided as a single file composed of four columns. The first two columns are the interacting nodes, the third column is the probability that the interaction is true, and the fourth column indicates whether the interaction is (D) or undirected (U). The fourth column is optional, and if not specified, all interactions are considered undirected. For the directed interactions, the direction is from the first node to the second. For instance, if the interaction is a kinase-to-substrate interaction, the direction is from kinase (first column) to substrate (second column). The user is free to use other protein interaction databases [[5-11](#_ENREF_5)] for human and other species.

EGFR GRB2 0.98 U

GRB2 SOS1 0.95 D

1. **Preparing the configuration files**

Both garnet.py and forest.py require configuration files. We provide samples of the garnet and forest configuration files in the example directories, with the .cfg file suffix.

1. **Prepare the garnet configuration file**. The garnet configuration file has 13 different variables provided as follows and is used to run a series of sub-scripts to identify transcription factors from gene expression data.

[chromatinData]

bedfile=*[bed file of accessible chromatin regions]*

fastafile=*[fasta file of same regions, collected via galaxyweb]*

genefile=*[path to garnet]/data/ucsc_hg19_knownGenes.txt*

xreffile=*[path to garnet]/data/ucsc_hg19_kgXref.txt*

windowsize=*[distance around transcription start site]*

[motifData]

tamo_file=*../data/matrix_files/vertebrates_clustered_ic8_motifs.tamo*

genome=hg19

numthreads=4

doNetwork=False

tfDelimiter=.

[expressionData]

expressionFile=*[name of expression file]*

pvalThresh=0.05

qvalThresh=0.05

Here, all the input files have to be given with their full paths. bedfile reflects regions of open chromatin, or promoter regions, to be scanned. fastafile is the fasta sequences of regions from bedfile (see Step 4).

The genefile, xreffile map genome coordinates to genes and gene identifiers to gene symbols (respectively) and are provided in cases where the user is using the hg19 or mm9 builds of the UCSC genome. Otherwise they can be downloaded from the UCSC website. The tamo_file is also provided, and the windowsize reflects the distance to look from transcription start site (TSS).

The [expressionData] segment of the configuration file is optional, as garnet will still run without gene expression data. In that case, it will score all potential transcription factor-DNA interactions without selecting the most likely transcription factor. The expressionFile contains the gene expression data as explained at Step 3, and the pvalThresh and qvalThresh determine how to select transcription factorsfor formatting into the events_to_genes_with_motifsregression_results_FOREST_INPUT.xls file. qvalThresh will only be used if pvalThresh is blank.

1. **Prepare the forest configuration file.** The required parameters in the configuration file are ω (artificial edge cost), β (scaling factor for node prizes), µ (optional scaling factor of negative prizing), and D (depth of the final network). See Table 2 for the details of all these parameters, as well as other optional command-line arguments and how to tune them or the README.md file in the Omics Integrator package.

w = 1

b = 1

D = 10

mu = 0.1

1. **Running garnet and forest**

Garnet and Forest can be run independently or together. To interpret gene expression data in the network context, you must run garnet.py prior to forest.py. We recommend starting with one of the examples provided in the example/ directory to begin your analysis. example/a549/test-tgfb-data.py showcases all of the features of Garnet and F­orest.

- 1. Run garnet.py. Timing ~1-5+ hours.

$ cd garnet-forest-0.0.1/scripts

$ python garnet.py [config_file_name] --outdir=[outdir]

Garnet will create a directory named outdir and place the results in that directory.

- 1. Run forest.py. Timing ~1-20 minutes

Forest will not create a directory for output, so you can either create one yourself or use the same directory that was used to run garnet.py.

$ python forest.py --prize ../example/a549/Tgfb_phos.txt --edge ../data/iref_mitab_miscores_2013_08_12_interactome.txt --conf ../example/a549/Tgfb_forest.cfg --dummyMode terminals --outpath outdir --outlabel protocol_results

The details of the options to run the script are described in **Table 2**. To see the help, type

$ python forest.py –h

Forest can also be automated with numerous parameters. For an example of running forest within a script, see example/a549/test-tgfb-data.py.

1. **Visualization of the results.**

To visualize the network in Cytoscape, open Cytoscape, select File > Import > Network > File..., select the .sif format network file to open and click ‘OK’. To view the node and edge attributes, select File > Import > Table > File... and select the result_nodeattributes.tsv or the result_edgeattributes.tsv. The user can adapt the visualization of the nodes and edges using attributes files and alter the layout of the network.

**3. Output Files Created by Garnet and Forest**

Both Garnet and Forest run a series of scripts to perform their associated analysis. Here we describe the files in the order in which they are created to both facilitate debugging and also improve understanding of how the algorithms work.

1. Files produced by Garnet

Garnet produces a series of intermediate files while scanning the epigenetic data for transcription factor binding motifs. These files will be placed in the directory provided by the --outdir option.

1-[dirname]/events_to_genes.fsa This file contains the regions of the fastafile provided in the configuration file that are within the specified distance to a transcription start site.

2-[dirname]/events_to_genes.xls: This file contains an entry for each epigenetic region, including the coordinates for that region, the epigenetic activity in that region, and the relationship of that region to the closest gene.

3-[dirname]/events_to_genes_with_motifs.txt: This contains the raw transcription factor scoring data for each region in the fasta file.

4-[dirname]/events_to_genes_with_motifs.tgm: This contains the best score for each transcription factor matrix mapped to each gene.

5-[dirname]/events_To_genes_with_motifs_tfids.txt: Names of transcription factors (or columns) of the matrix.

6- [dirname]/events_to_genes_with_motifs_geneids.txt: Names of genes (or rows) of the matrix.

7-[dirname]/events_to_genes_with_motifs.pkl: A Pickle-compressed Python File containing a dictionary data structure that contains files 4-6 (under the keys ‘tgm’,’tfs’, and ‘genes’) respectively as well as a ‘delim’ key that describes what delimiter was used to separate out TFs in the case where there are multiple TFs in the same family.

8-[dirname]/events_to_genes_with_motifsregression_results.xls: Results from linear regression.

9-[dirname]/events_to_genes_with_motifsregression_results_FOREST_INPUT.xls: Only those results from the regression that fall under a provided significance threshold, e.g. p=0.05. This file can be used as input to Forest under the --garnet option.

1. Files produced by Forest

Forest produces multiple result files that enable the user to view the output in different contexts. Forest uses an output directory dirname according to the --outpath argument and names the files label according to the --outlabel argument.

1-[dirname]/[label]_info.txt: This file contains information about the execution of the msgsteiner code and any errors that might have arisen.

1-[dirname]/[label]_optimalForest.sif: This file contains the optimal output forest, which excludes edges to the dummy node.

2-[dirname]/[label]_dummyForest.sif: This file contains the optimal network output before removing the dummy node. This file is useful for debugging, as networks that contain many trees with only one node are not desirable.

3-[dirname]/[label]_augmentedForest.sif: This file contains the nodes from the optimal network output connected by all the interactions that were present between them in the original interactome.

4-[dirname]/[label]_edgeattributes.tsv: A tab-separated value file containing information for each edge in the network, including the weight in the interactome, and the fraction of optimal networks containing this edge in the case where network randomization was performed, i.e. from adding noise to the prizes and re-running the algorithm several times. In cases where the user selects the –cyto28 option, this file will be divided into individual files for each attribute.

5-[dirname]/[label]_nodeattributes.tsv: A tab-separated value file containing information for each node in the network, such as the prize (p’(v)) and betweenness centrality in the augmented network. In cases where the user selects the –cyto28 option, this file will be divided into individual files for each attribute.

The network files can be visualized in Cytoscape, as described in the text.

**Test Dataset**

The expression and phosphoproteomic data have been retrieved from the work by Thomson et al., 2011 [[12](#_ENREF_12)]. The epigenetic data have been downloaded from ENCODE [[13](#_ENREF_13)]. The direct links to these data are <ftp://hgdownload.cse.ucsc.edu/goldenPath/hg19/encodeDCC/wgEncodeUwDnase/wgEncodeUwDnaseA549PkRep1.narrowPeak.gz> and <ftp://hgdownload.cse.ucsc.edu/goldenPath/hg19/encodeDCC/wgEncodeUwDnase/wgEncodeUwDnaseA549PkRep2.narrowPeak.gz>

**4. Troubleshooting**

**Garnet**

To handle the diversity of computational tasks required by Garnet, the script runs a series of shorter scripts that handle each task independently. Before Garnet runs a script, it ensures that the previous task has been run by searching for the necessary files. As Garnet reads the configuration file and populates the commands, it will print the individual script commands to the screen. Therefore, if a command fails to run, Garnet will terminate and the user can re-run the sub-script with different parameters depending on the error.

There are several troubleshooting steps to follow if Garnet fails to run. First, check to ensure that all files in the configuration file have the full file path and exist. Next, ensure that the files are properly formatted – there are sample bed and fasta files in the example/ directory and sample gene mapping files in the data/ directory. If no significant transcription factors are selected, you can increase the pvalThresh parameter used to select factors of interest.

**Forest**

Forest will print warnings whenever it comes across something unexpected and corrects problems automatically when possible. Edge weights that are not between 0 and 0.99 will be noted and set to a maximum weight of 0.99. Self-edges from a protein to itself are removed from the interactome. Forest also prints a warning if a large percentage of the names in the prize file do not have matches in the interactome listed in the edge file because these proteins will not be included in the output network. This error may result from using two different naming schemes for the proteins or using the wrong interactome, in which case the user should use an external name conversion tool [[14](#_ENREF_14),[15](#_ENREF_15)] to map all entity names to the same type of identifier.

There are several troubleshooting steps to follow if Forest returns an empty network. First, verify that a sufficient number of terminals are present in the interactome. Next, try varying the input parameters that directly influence the size of the output network. Increasing β tends to produce larger output networks because the algorithm will prefer to connect more terminal nodes even if doing so requires using costlier edges. If µ is too large it can also lead to empty networks because the terminals may have small or negative prizes after correcting for degree (Equation 2) and potential Steiner nodes may have prizes that are so negative that they outweigh the benefits of connecting additional prizes. If ω is too large then it will not be possible to find a forest that scores better than an empty network because the cost of connecting the forest to the artificial node v_0_ will dominate the benefits of collecting the prizes in the forest.

**References**

1. Shannon P, Markiel A, Ozier O, Baliga NS, Wang JT, et al. (2003) Cytoscape: a software environment for integrated models of biomolecular interaction networks. Genome Res 13: 2498-2504.

2. Zhang Y, Liu T, Meyer CA, Eeckhoute J, Johnson DS, et al. (2008) Model-based analysis of ChIP-Seq (MACS). Genome Biol 9: R137.

3. Guo Y, Papachristoudis G, Altshuler RC, Gerber GK, Jaakkola TS, et al. (2010) Discovering homotypic binding events at high spatial resolution. Bioinformatics 26: 3028-3034.

4. Srikanth C, Wall DM, Maldonado-Contreras A, Shi HN, Zhou D, et al. (2010) Salmonella pathogenesis and processing of secreted effectors by caspase-3. Science 330: 390-393.

5. Razick S, Magklaras G, Donaldson IM (2008) iRefIndex: a consolidated protein interaction database with provenance. BMC Bioinformatics 9: 405.

6. Licata L, Briganti L, Peluso D, Perfetto L, Iannuccelli M, et al. (2012) MINT, the molecular interaction database: 2012 update. Nucleic Acids Res 40: D857-861.

7. Chatr-Aryamontri A, Breitkreutz BJ, Heinicke S, Boucher L, Winter A, et al. (2013) The BioGRID interaction database: 2013 update. Nucleic Acids Res 41: D816-823.

8. Franceschini A, Szklarczyk D, Frankild S, Kuhn M, Simonovic M, et al. (2013) STRING v9.1: protein-protein interaction networks, with increased coverage and integration. Nucleic Acids Res 41: D808-815.

9. Isserlin R, El-Badrawi RA, Bader GD (2011) The Biomolecular Interaction Network Database in PSI-MI 2.5. Database (Oxford) 2011: baq037.

10. Keshava Prasad TS, Goel R, Kandasamy K, Keerthikumar S, Kumar S, et al. (2009) Human Protein Reference Database--2009 update. Nucleic Acids Res 37: D767-772.

11. Pagel P, Kovac S, Oesterheld M, Brauner B, Dunger-Kaltenbach I, et al. (2005) The MIPS mammalian protein-protein interaction database. Bioinformatics 21: 832-834.

12. Thomson S, Petti F, Sujka-Kwok I, Mercado P, Bean J, et al. (2011) A systems view of epithelial-mesenchymal transition signaling states. Clin Exp Metastasis 28: 137-155.

13. Consortium EP (2004) The ENCODE (ENCyclopedia Of DNA Elements) Project. Science 306: 636-640.

14. Haider S, Ballester B, Smedley D, Zhang J, Rice P, et al. (2009) BioMart Central Portal--unified access to biological data. Nucleic Acids Res 37: W23-27.

15. Huang da W, Sherman BT, Lempicki RA (2009) Systematic and integrative analysis of large gene lists using DAVID bioinformatics resources. Nat Protoc 4: 44-57.
